# The emerging role of perivascular cells (pericytes) in viral pathogenesis

**DOI:** 10.1099/jgv.0.001634

**Published:** 2021-08-23

**Authors:** Teemapron Butsabong, Mariana Felippe, Paola Campagnolo, Kevin Maringer

**Affiliations:** ^1^​ Department of Biochemical Sciences, Faculty of Health and Medical Sciences, University of Surrey, Guildford, Surrey, GU2 7XH, UK; ^2^​ The Pirbright Institute, Pirbright, Surrey, GU24 0NF, UK

**Keywords:** blood-brain barrier, COVID-19, dengue haemorrhage, HIV/AIDS, pericyte, SARS-CoV-2

## Abstract

Viruses may exploit the cardiovascular system to facilitate transmission or within-host dissemination, and the symptoms of many viral diseases stem at least in part from a loss of vascular integrity. The microvascular architecture is comprised of an endothelial cell barrier ensheathed by perivascular cells (pericytes). Pericytes are antigen-presenting cells (APCs) and play crucial roles in angiogenesis and the maintenance of microvascular integrity through complex reciprocal contact-mediated and paracrine crosstalk with endothelial cells. We here review the emerging ways that viruses interact with pericytes and pay consideration to how these interactions influence microvascular function and viral pathogenesis. Major outcomes of virus-pericyte interactions include vascular leakage or haemorrhage, organ tropism facilitated by barrier disruption, including viral penetration of the blood-brain barrier and placenta, as well as inflammatory, neurological, cognitive and developmental sequelae. The underlying pathogenic mechanisms may include direct infection of pericytes, pericyte modulation by secreted viral gene products and/or the dysregulation of paracrine signalling from or to pericytes. Viruses we cover include the herpesvirus human cytomegalovirus (HCMV, *Human betaherpesvirus 5*), the retrovirus human immunodeficiency virus (HIV; causative agent of acquired immunodeficiency syndrome, AIDS, and HIV-associated neurocognitive disorder, HAND), the flaviviruses dengue virus (DENV), Japanese encephalitis virus (JEV) and Zika virus (ZIKV), and the coronavirus severe acute respiratory syndrome-related coronavirus 2 (SARS-CoV-2; causative agent of coronavirus disease 2019, COVID-19). We touch on promising pericyte-focussed therapies for treating the diseases caused by these important human pathogens, many of which are emerging viruses or are causing new or long-standing global pandemics.

The vascular system serves every tissue within the body and performs numerous life-critical functions, including the transport of nutrients, gases, hormones and other signalling molecules, immune cells and humoral mediators of immunity. A number of viruses replicate in vascular cells and/or immune cells circulating within the vascular system. In addition, the ability to enter the bloodstream and establish viraemia is essential for the transmission of many viruses, most notably blood-borne viruses and arthropod-borne viruses (arboviruses), while other viruses exploit the vascular system for dissemination from the primary site of infection to other target organs. In some cases, the disruption of vascular integrity and/or function directly contributes to viral pathogenesis, for example by mediating haemorrhage or neurotropic symptoms.

Pericytes, a type of perivascular cell, have recently emerged as key players in the pathogenesis of human viral pathogens from diverse taxa (Table 1, Fig. 1). We here summarise the emerging contribution that pericytes play in viral pathogenesis. Broader interactions between viruses and the cardiovascular system were recently reviewed elsewhere [1].

**Fig. 1.**
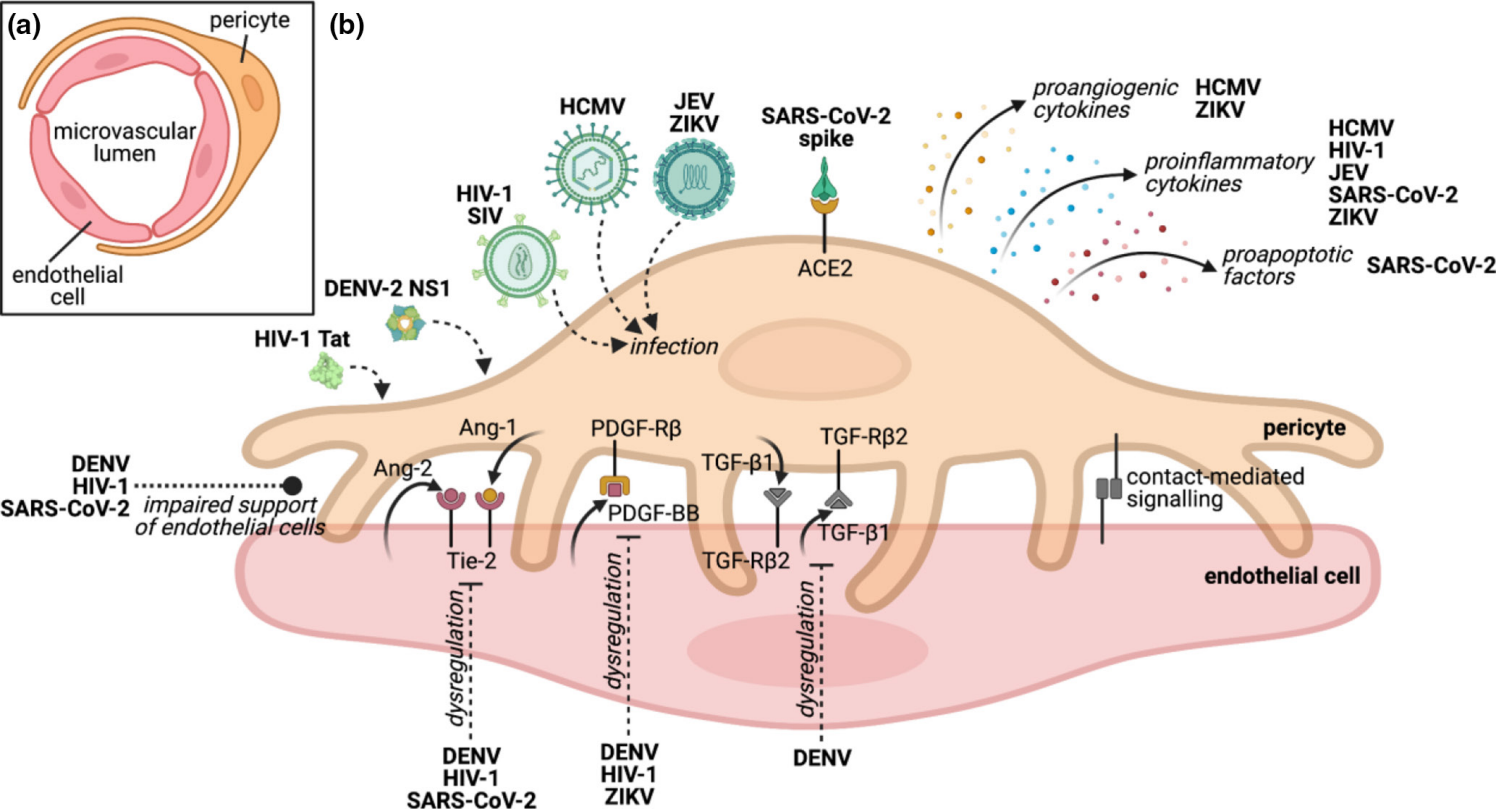
Modulation of pericyte functions by viruses. (**a**) Cross-section of microvascular architecture. (**b**) Pericytes support endothelial barrier formation and maintenance via contact-mediated interactions and paracrine signalling with endothelial cells. Uni- and bi-directional paracrine signalling and contact-mediated molecular interactions important for pericyte and endothelial cell function and relevant to viral infections discussed here are shown. Signalling molecules are not necessarily unique to pericytes or endothelial cells, and viral modulation thereof may additionally involve other cell types. Impacts of viruses on pericytes indicated in italics. The cellular receptors bound by DENV-2 NS1 and HIV-1 Tat remain unknown. ACE2, angiotensin-converting enzyme 2; Ang-1/–2, angiopoietin 1/2; DENV, dengue virus; HCMV, human cytomegalovirus; HIV-1, human immunodeficiency virus type 1; JEV, Japanese encephalitis virus; NS1, nonstructural protein 1; PDGF-BB, platelet-derived growth factor BB; PDGF-Rβ, PDGF receptor beta; SARS-CoV-2, severe acute respiratory syndrome-related coronavirus 2; SIV, simian immunodeficiency virus; Tat, transactivator of transcription; TGF-β1, transforming growth factor beta 1; TGF-Rβ2, TGF receptor beta 2; Tie-2, tyrosine kinase with immunoglobulin-like epidermal growth factor (EGF)-like domains 2; ZIKV, Zika virus. Illustration created with BioRender.com; Tat image rendered using data from Protein Data Bank (PDB, www.rcsb.org, 3D structure reference 1TIV) [89, 90].

**Table 1.** Summary of known virus-pericyte interactions.

Family (Subfamily)	Genus	Species	Pericyte interaction	References
*Coronaviridae* (*Orthocoronavirinae*)	*Betacoronavirus*	*Severe acute respiratory syndrome-related coronavirus 2* (SARS-CoV-2)	Reduced pericyte coverage in alveolar capillaries *in vivo* Dysregulated angiopoietin signalling *in vivo* Reduced endothelial cell support, increased migration and secretion of proinflammatory and proapoptotic factors in cardiac pericytes treated with spike protein *in vitro*	[58] [81] [57]
*Flaviviridae*	*Flavivirus*	*Dengue virus* (DENV)	Soluble NS1-mediated reduction in pericyte-endothelial cell interaction and increased coculture permeability *in vitro* Dysregulated angiopoietin signalling and reduced serum PDGF-BB and TGF-β correlating with severe dengue *in vivo*	[64] [68–70, 73]
		*Japanese encephalitis virus* (JEV)	Infection of brain pericytes *in vitro* (rat) and *in vivo* (mouse) Proinflammatory cytokine secretion in infected pericytes *in vitro* (rat) and *in vivo* (mouse)	[49] [49]
		*Zika virus* (ZIKV)	*In vitro* infection of brain and retinal pericytes *In vivo* infection of brain pericytes (mouse) Proinflammatory and angiogenic cytokine secretion in infected pericytes *in vitro* Reduced levels of serum PDGF-BB *in vivo*	[46, 47] [46] [47] [74]
*Herpesviridae* (*Betaherpesvirinae*)	*Cytomegalovirus*	*Human betaherpesvirus 5* (human cytomegalovirus, HCMV)	*In vitro* infection of brain, placental, retinal and inner blood-retinal barrier pericytes *In vivo* infection of brain and placental pericytes *In vitro* and *in vivo* infection of renal mesangial cells^1^ and perivascular mesenchymal stromal cells^2^ in brain, liver, lung and bone marrow Placental pericyte loss *in vivo* Proinflammatory and angiogenic cytokine secretion in infected pericytes *in vitro* and *in vivo*	[23–25] [23, 25] [26, 27] [25] [23–25]
*Retroviridae* (*Orthoretrovirinae*)	*Lentivirus*	*Human immunodeficiency virus 1* (HIV-1)	*In vitro* infection of brain and lung pericytes Brain pericyte loss and blood-brain barrier destabilisation *in vivo* Infection-associated increase in pericyte-endothelial cell coculture permeability *in vitro* Pericyte-dependent enhancement of blood-brain barrier endothelium penetration by cell-free HIV-1 *in vitro* Proinflammatory cytokine secretion, cytoskeleton remodelling and modulation of extracellular matrix and adhesion proteins in infected pericytes *in vitro* Soluble Tat-mediated increase in migration, PDGF-BB expression and PDGF-Rβ activation in brain pericyte *in vitro* Dysregulated angiopoietin signalling and increased PDGF-BB expression *in vivo*	[35–38, 43] [31–35] [37] [39] [31, 32] [34] [34, 75–77]
		*Simian immunodeficiency virus* (SIV)	Brain pericyte loss and blood-brain barrier destabilisation *in vivo* (rhesus macaque) Infection of lung pericytes *in vivo* (rhesus and cynomolgus macaques)	[33] [43]

Model organism species is indicated only where human data is unavailable. NS1, nonstructural protein 1; PDGF-BB, platelet-derived growth factor BB; PDGF-Rβ, PDGF receptor beta; Tat, transactivator of transcription; TGF-β, transforming growth factor beta.

*Renal mesangial cells are a tissue-specific pericyte lineage.

†Perivascular mesenchymal stromal cells are a cell population closely related to pericytes.

## The role of pericytes in microvascular barrier formation and maintenance

Endothelial cells line the lumen of the vasculature throughout the body and form the primary barrier within the vascular system. Large vessels such as arteries and veins are composed of a single endothelial cell layer (tunica intima) surrounded by multiple layers of smooth muscle cells (tunica media), a highly contractile type of perivascular cells, and a fibrous layer of loose collagenous matrix and embedded cells (tunica adventitia). In small blood vessels, such as capillaries and post-capillary venules, the endothelial layer is ensheathed by less mature perivascular cells (pericytes). The perivascular space surrounding the capillaries and the tunica adventitia in the large vessels contain several other subpopulations of cells actively contributing to vascular function. A typical example are perivascular immune cells such as macrophages and neural microglia, which contribute to immune responses and pathogen clearance, and also regulate vascular permeability and function [2, 3]. Additionally, perivascular fibroblasts and mesenchymal progenitor cells produce extracellular matrix and contribute to the pool of perivascular cells [4].

Pericytes of the microvasculature are large mesenchymal-like cells of irregular shape with long finger-like projections that form close physical contacts with multiple endothelial cells, maintaining and supporting vascular homeostasis and barrier function (Fig. 1a) [5–7]. Pericyte-dependent barrier control is exerted both through close physical contact with endothelial cells and through reciprocal paracrine signalling [5]. Pericytes also express receptors for a number of inflammatory mediators and respond to these and other pathological stimuli (e.g. lipopolysaccharide, hypoxia and high glucose) by regulating vascular function. Pericytes regulate vascular barrier function both directly, for example through contraction or by increasing capillary permeability, and indirectly by modulating the phenotype of effector cells, such as microglia, and attracting immune cells through the release of soluble mediators [8–10]. As well as responding to pathological stimuli, pericytes themselves exhibit macrophage-like activities such as phagocytosis, and act as antigen-presenting cells by displaying antigens through major histocompatibility complex class II (MHC-II) [10]. Pericytes also drive vascular regeneration through the regulation of angiogenesis. During this process, newly formed endothelial sprouts attract the migration and adhesion of pericytes, which in turn stabilise the new vessels. Importantly, pericytes are considered progenitor cells with pluripotent capacities and have been exploited in regenerative medicine and tissue engineering applications, leveraging their differentiation capacity and pro-angiogenic function [11].

The interaction between endothelial cells and pericytes is critical to the maintenance of vascular function and is mediated both by contact-dependent signalling and by soluble factors [12]. Reciprocal paracrine signalling between the two cell types is mediated by receptors that are in many cases uniquely present either on endothelial cells or on pericytes, and these receptors respond to stimuli produced uniquely by the counterpart cell type within this two-cell partnership (Fig. 1b), though some of these signalling molecules are also produced by other cells in the body [12]. A typical example of this close paracrine interaction is platelet-derived growth factor BB (PDGF-BB), which is produced by endothelial cells during angiogenesis to promote the recruitment of pericytes, which express the PDGF receptor beta (PDGF-Rβ) [12]. In addition, endothelial cells express the receptor Tie-2 (tyrosine kinase with immunoglobulin-like epidermal growth factor (EGF)-like domains 2) which is stimulated by its pericyte-secreted ligand angiopoietin 1 (Ang-1), contributing to blood vessel stability and maturation [12]. Meanwhile, endothelial cells secrete the autocrine antagonist Ang-2, which competes with Ang-1 for the Tie-2 receptor to provide fine regulation of vessel permeability [12]. Finally, transforming growth factor beta (TGF-β) and TGF receptors (TGFR) are expressed by both endothelial cells and pericytes and are crucial for the formation of pericyte coverage around endothelial cells [12].

Despite their patent organ specialisation within the adult body, all endothelial cells share a single embryonic origin [13]. Instead, perivascular cells in different organs have distinct embryonic origins, and pericytes have also been proposed to derive from tissue-resident mesenchymal cells and constitute a tissue-resident progenitor cell reservoir [12], though this remains somewhat controversial. These tissue-specific origins explain at least in part the nuanced differences in pericyte roles and functions in different organs. For example, in the blood-brain barrier, the high density of pericytes and their close association with endothelial cells and astrocytes is critical for the maintenance of selective tight-junctions and barrier integrity [14]. In contrast, in the liver, pericytes are commonly referred to as hepatic stellate cells (HSC) and store the majority of the vitamin A in the body and have an important role in liver regeneration and fibrosis following injury or a partial resection [15]. Even within the same tissue, several subpopulations of pericytes characterised by differential marker expression exist. In some cases, these distinct subpopulations display different differentiation patterns and perform distinct functions [16–18]. For example, pericytes in the adipose tissue are classified into two different subpopulations characterised by the expression of either CD146 or CD34. While both populations exhibit similar localisation and express other pericyte markers equally, CD146+ pericytes are osteogenic while CD34+ pericytes mainly secrete pro-angiogenic signals [19].

A number of noncommunicable diseases are characterised by an abnormal pericyte to endothelial cell ratio, including stroke, multiple sclerosis, diabetes and various tumours [12, 14]. Loss of pericytes in the brain can cause neurodegenerative and neuroinflammatory diseases [20]. In diabetic retinopathy, a significant loss of retinal pericytes results in acellular capillaries, and ultimately hyperpermeability, microaneurysms and uncontrolled endothelial cell proliferation that lead to blindness [21]. Understanding potential similarities in pericyte dysfunction in noncommunicable and infectious pathologies may lead to new methods of diagnosing and treating viral diseases.

## Direct viral infection of pericytes

A number of viruses are able to infect pericytes, including human cytomegalovirus (HCMV, *Human betaherpesvirus 5*), human immunodeficiency virus (HIV), Japanese encephalitis virus (JEV) and Zika virus (ZIKV). Pericyte infection has most often been demonstrated in pericytes of the blood-brain barrier, suggesting a common role for pericytes in the neuropathogenesis of these viruses. A frequent outcome of pericyte infection is the modulation of inflammatory responses, suggesting that pericytes should not be overlooked when considering the wider contribution of immune dysfunction to viral pathogenesis.

### HCMV

The beta-herpesvirus HCMV causes life-long persistent infection, with a seroprevalence of 60–100% depending on location and socioeconomic factors [22]. Although infections are usually asymptomatic in immunocompetent adults, HCMV is a common opportunistic pathogen in acquired immunodeficiency syndrome (AIDS) and organ transplantation, while intrauterine infection with HCMV is the leading infectious cause of congenital abnormalities in the developed world [22]. Pericytes from the brain, retina and inner blood-retinal barrier support HCMV infection and replication *in vitro*, resulting in pericyte cell death, and are more permissive for HCMV than other cell types found in the brain and retinal neurovascular bed [23, 24]. *In vivo*, HCMV infection of brain pericytes was detected in an HIV-infected patient with HCMV-associated neuropathology [23]. Furthermore, infection of brain and retinal pericytes *in vitro* was found to induce the secretion of proinflammatory cytokines, including interleukin 1β (IL-1β), IL-6, IL-8, RANTES, macrophage inflammatory protein 1α (MIP-1α) and interferon-inducible T-cell alpha chemoattractant (I-TAC), suggesting that pericyte infection may contribute to HCMV-associated neuroinflammation and retinal dysfunction, and hence loss of vision and cognitive impairment in neonates [23, 24]. In addition, HCMV infects placental pericytes *in vitro* and *in vivo*, resulting in pericyte loss and vascular abnormalities *in vivo* and the induction of proinflammatory cytokines (IL-6; monocyte chemotactic protein 1, MCP-1) and angiogenic cytokines *in vitro*, which may contribute to viral dissemination, placental inflammation, and dysregulation of placental angiogenesis [25]. HCMV infection is also a cause of morbidity and mortality among solid organ (e.g. kidney) and haematopoietic stem cell transplant patients. In this context, the discovery that renal mesangial cells (a tissue-specific pericyte lineage), as well as perivascular mesenchymal stromal cells (a cell population closely related to pericytes) in the brain, liver, lung and bone marrow, support HCMV infection *in vitro* and *in vivo [26, 27]*, suggests that pericytes may also play some role in post-transplant complications.

### HIV

The retrovirus HIV has a world-wide distribution, with the largest burden of disease in sub-Saharan Africa [28]. HIV is the causative agent of AIDS, as well as HIV-associated neurocognitive disorder (HAND) [29]. HAND is associated with virus-induced pathology in the brain, spinal cord and peripheral nerves, which contributes to neurological and cognitive impairment [29]. An estimated 38 million people are currently living with HIV infection [30], with some degree of cognitive impairment observed in half of all patients undergoing antiretroviral therapy [29]. A reduction in the pericyte to endothelial cell ratio and consequent destabilisation of the blood-brain barrier have been observed *in vivo* in mouse models and in biopsies from HIV-positive patients, as well as following simian immunodeficiency virus (SIV) infection of rhesus macaques [31–35]. Furthermore, HIV-1 infects human brain pericytes *in vitro* via its receptor CD4 (cluster of differentiation 4) and co-receptors C-X-C chemokine receptor type 4 (CXCR4) and C-C chemokine receptor type 5 (CCR5), and replicates to low levels in these cells [35–38]. Infection of brain pericytes with HIV-1 *in vitro* remodels the cytoskeleton, increases the expression of adhesion proteins (including intercellular adhesion molecule-1, ICAM-1, and vascular cell adhesion molecule-1, VCAM-1) and of the proinflammatory cytokine IL-6, and reduces the production of extracellular matrix proteins (e.g. fibronectin and nidogen) [31, 32]. In addition, treatment of pericytes with cytokines that are typically overexpressed during HIV infection (including tumour necrosis factor alpha, TNF-α, and IL-1β) reduces their expression of PDGF-Rβ, rendering them less responsive to migratory stimuli, and increases proinflammatory gene expression [31, 32, 36, 38]. Consequently, infected or chronic inflammation-exposed pericytes are less able to support barrier integrity in pericyte-endothelial cell cocultures *in vitro* [37]. HIV is able to cross the blood-brain barrier as cell-free virus and within infected immune cells, allowing the virus to cause further neurological effects and evade antiretroviral therapy [39–42]. Interestingly, pericytes enhance the ability of cell-free HIV-1 to traverse the endothelial barrier in blood-brain barrier coculture models *in vitro* [39]. Overall, pericytes may therefore play an important role in facilitating the neurological manifestations of HIV infection. Furthermore, a recent report demonstrating infection of human lung pericytes with HIV *in vitro*, and infection of macaque lung pericytes with SIV *in vivo*, suggests that pericytes may play an underappreciated wider role in HIV pathogenesis [43].

### ZIKV

The flavivirus ZIKV can cause developmental disorders, including microcephaly, while causing generally mild symptoms in otherwise healthy adults [44]. ZIKV is endemic in Africa and Asia and caused a major pandemic following its introduction into the Americas in 2015 [45]. In adult interferon-deficient footpad-infection mouse models, ZIKV was found to infect pericytes in the choroid plexus and meninges before spreading to the cortex [46]. Furthermore, ZIKV infects human brain vascular pericytes *in vitro*, causing an increase in endothelial barrier permeability, suggesting that pericyte infection may facilitate entry of ZIKV into the central nervous system [46]. ZIKV has also been found to infect human retinal pericytes and endothelial cells of the inner blood-retinal barrier *in vitro* [47]. The associated induction of proinflammatory cytokine RANTES and angiogenic factors was proposed to contribute to congenital ocular disease, which is commonly observed in microcephalic infants following ZIKV infection [47].

### JEV

The flavivirus JEV is the leading cause of viral encephalitis in Southeast Asia, China and the Western Pacific, causing 50 000 to 70 000 symptomatic cases and 10 000 deaths each year [48]. JEV was shown to infect rat brain microvascular pericytes *in vitro*, inducing the secretion of proinflammatory cytokines that increased the permeability of primary rat endothelial cell monolayers [49]. Furthermore, the pericyte marker PDGF-Rβ mediates JEV infection and the tyrosine kinase inhibitor Imatinib, which also inhibits PDGF-Rβ, reduces JEV infection-associated brain pathology and overall lethality *in vivo* [50]. Moreover, infection of brain perivascular cells by JEV, and an associated induction of proinflammatory cytokines (IL-6 and RANTES) was confirmed *in vivo* in mice [49]. The shared tropism of both JEV and ZIKV for neurovascular pericytes may be indicative of a wider role for pericytes in facilitating the entry of neuropathogenic flaviviruses into the central nervous system.

### Severe acute respiratory syndrome-related coronavirus 2 (SARS-CoV-2)

The beta-coronavirus SARS-CoV-2 emerged in China in 2019 and rapidly spread across the world, causing the coronavirus disease 2019 (COVID-19) pandemic [51]. Although infections with SARS-CoV-2 are generally asymptomatic or present with mild respiratory symptoms, older individuals and those with comorbidities such as cardiovascular disease, diabetes, chronic respiratory disease or obesity can develop life-threatening respiratory pathologies, cardiovascular complications and multiorgan failure [51, 52]. Direct infection of pericytes with SARS-CoV-2 has not been reported. However, angiotensin-converting enzyme 2 (ACE2), the entry receptor for SARS-CoV-2 [51], is highly expressed in mouse olfactory pericytes and human cardiac pericytes [53–56]. Interestingly, a recent preprint showed that treatment of human cardiac pericytes with purified recombinant SARS-CoV-2 spike protein, the primary receptor-binding protein in the SARS-CoV-2 virion, induces functional changes including increased migration, reduced support of endothelial cells, and the secretion of proinflammatory cytokines (IL-1β, IL-6, TNF-α and MCP-1) and proapoptotic factors [57]. Furthermore, pericyte coverage has been found to be reduced in alveolar capillaries in COVID-19 patients [58]. It has therefore been proposed that pericyte infection may contribute to the cardiovascular-respiratory symptoms of COVID-19.

## Impact of secreted viral proteins on pericytes

### HIV

Several viruses secrete viral gene products into the extracellular space and these viral proteins are able to mediate disseminated effects independently of viral infection. For example, the HIV-1 transactivator of transcription (Tat) is secreted from infected cells and has been implicated in the development of HAND [34]. Treatment of primary human brain vascular pericytes and the pericyte-like C3H/10T1/2 cell line with recombinant soluble Tat was shown to increase cell migration *in vitro* [34]. Furthermore, pericyte coverage is reduced in brain microvessels both in biopsies from patients with HIV encephalitis and in transgenic (Tg26) mice expressing Tat and other viral gene products from an HIV-1 proviral genome lacking the *gag* and *pol* genes [34], suggesting that Tat-induced pericyte dysfunction and/or loss may contribute to the progression of HAND.

### Dengue virus (DENV)

The mosquito-borne flavivirus DENV is the most significant arthropod-borne virus (arbovirus) of humans, infecting an estimated 390 million people every year across the tropics and subtropics [59]. Severe dengue disease is potentially fatal and is typified by multiorgan microvascular hyperpermeability and circulatory shock; the disease has multifactorial causes that are at least partially mediated by the viral nonstructural protein 1 (NS1) [60, 61]. NS1 is a glycoprotein secreted into the circulatory system as a hexamer and has been shown to induce permeability in primary human endothelial cell monolayers from a variety of organs *in vitro* by altering the endothelial glycocalyx [62, 63]. In addition, we recently demonstrated that the *in vitro* effects of DENV-2 NS1 on endothelial cells are more pronounced in the presence of primary human pericytes [64]. Treatment with recombinant soluble NS1 disrupted pericyte-endothelial cell interactions in 3D cocultures and increased barrier permeability, without affecting pericyte migration capacity or viability [64]. These effects were mediated at least partially by the disruption of contact-independent paracrine signalling between pericytes and endothelial cells [64]. Our findings suggest that pericytes may play an as-yet underappreciated role in dengue vascular leakage.

## Viral modulation of pericyte-relevant paracrine signalling

Given the myriad ways in which viruses interact with pericytes, and the ease with which vascular signalling molecules can be measured in patient blood, it is not surprising that serological changes suggestive of dysregulated pericyte-endothelial cell signalling have been detected during viral infection. Angiopoietin levels, and in particular the balance between Ang-1 and Ang-2, are critical in the maintenance of the vascular barrier [12]. Although angiopoietin dysregulation has been associated with a number of cancer-associated viruses, including hepatitis B virus (HBV), hepatitis C virus (HCV) and Kaposi’s sarcoma-associated herpesvirus (KSHV, *Human gammaherpesvirus 8*) [65], and pericytes are known to be dysregulated in the tumour environment [66, 67], we are unaware of data supporting a specific link to pericytes for these viruses and they are therefore not discussed further.

### DENV

A number of papers have correlated changes in serum angiopoietin levels with the severity of dengue disease [68–70]. Patients with severe dengue exhibit reduced Ang-1 levels and increased Ang-2 levels, and hence a significantly increased Ang-2/Ang-1 ratio, during the life-threatening critical stage of disease that is associated with vascular leakage and shock [68, 70]. Angiopoietin levels and the Ang-2/Ang-1 ratio return to baseline levels at the point of recovery and discharge from hospital [68, 69]. Interestingly, infection of both human dermal microvascular endothelial cells and human umbilical vein endothelial cells (large vessel-derived cells) with DENV results in a downregulation of Ang-1 and upregulation of Ang-2 expression and secretion *in vitro* [71, 72], mirroring the serological observations *in vivo*. Treatment of endothelial cells with recombinant soluble NS1 also enhances Ang-2 secretion *in vitro* [72]. Both DENV infection and NS1 treatment increase the permeability of endothelial cell monolayers *in vitro*, which is reversed by Ang-1 treatment [62, 63, 71, 72]. Dengue patients also exhibit reduced plasma levels of PDGF-BB and TGF-β in the acute phase of disease [73], and a reduction in PDGF-BB is also observed during infection with the related ZIKV [74]. How these observed serological differences affect, or may be influenced by, pericytes remains to be determined.

### HIV

The PDGF-BB/PDGF-Rβ system is affected by HIV infection and may modulate the enhanced HIV-associated neurological impacts observed in substance-abusing patients. PDGF-BB expression is upregulated in HIV-1-infected macrophages and Tat-treated primary human brain vascular pericytes, pericyte-like C3H/10T1/2 cells, smooth muscle cells and primary human pulmonary arterial endothelial cells *in vitro* [34, 75]. Furthermore, PDGF-BB expression is increased *in vivo* in Tg26 mice containing the HIV-1 proviral genome, in brain microvessels from HIV encephalitis patients, and in lung tissue from HIV-positive patients [34, 75]. PDGF-Rβ activation following Tat treatment was also demonstrated in pericytes *in vitro*, and this activation of the PDGF-BB/PDGF-Rβ axis was proposed to contribute to pericyte loss in the brain microvasculature in HAND [34]. Similarly to dengue, the serum of HIV-positive patients exhibits reduced Ang-1 and enhanced Ang-2 levels, which is mitigated by antiretroviral therapy [76, 77]. Reduced secretion of Ang-1, as well as TGF-β1, has also been observed in primary human brain vascular pericytes *in vitro* [31]. Taken together, these data suggest that the observed dysregulation of the Ang-1/Ang-2 and PDGF-BB/PDGF-Rβ systems in HIV-positive individuals may be at least partially influenced by pericytes.

### Other viruses

HCMV and SARS-CoV-2 also modulate pericyte-related signalling molecules, but the effects are less extensively characterised than for DENV or HIV. *In vitro* infection of smooth muscle cells (large vessel perivascular cells) or human umbilical vein endothelial cells with HCMV results in increased PDGF-Rβ or reduced Ang-2 expression respectively [78, 79]. HCMV-induced PDGF-Rβ upregulation and consequent uncontrolled growth of perivascular cells has been proposed to contribute to transplantation-associated restenosis, a common post-transplant complication triggered by the damage incurred to blood vessels during the procedure, for which HCMV infection status is a risk factor [79]. Meanwhile, elevated serum Ang-2 levels have been correlated with pulmonary disease severity and admission into intensive care in COVID-19 patients and clinical trials testing the potential therapeutic benefit of Ang-2 blockade are underway [80, 81], though no direct link to pericytes has as yet been reported.

It is important to note that the soluble signalling molecules discussed here are not exclusively secreted by pericytes or their endothelial cell partners. Furthermore, at least some of the impacts of viruses on pericytes are known to be mediated directly or indirectly via changes to contact-dependent signalling molecules such as tight junction proteins on pericytes and/or endothelial cells [35].

## Conclusions and future directions

The crucial role that pericytes play in maintaining vascular integrity is reflected in the varied and increasing number of ways in which pericyte modulation by viruses is being shown to contribute to pathogenesis. We anticipate that much remains to be discovered in this arena, especially because most of the existing literature on *in vitro* virus-microvascular interactions has focussed on endothelial cell monocultures that do not fully recapitulate complex and multicellular microvasculature structure. A number of viruses are known to infect endothelial cells, including DENV, Ebola virus (EBOV), several hantaviruses, HCMV, Hendra virus (HeV), human herpesvirus 6 (HHV-6), influenza A virus, Nipah virus (NiV) and SARS-CoV-2 [82]. How endothelial cell infection with these viruses may impact on pericyte function is largely unknown. Whether secreted viral gene products other than DENV-2 NS1 and HIV-1 Tat modulate pericyte function also remains to be determined. Another underexplored area relates to the diverse developmental origins of pericytes throughout the body, and the potentially diverse characteristics of pericyte subpopulations within a given tissue, which may underpin some of the tissue-specific microvascular dysfunction observed during viral diseases. Finally, even pericyte-endothelial cell coculture models neglect the important impacts that immune cell-derived cytokines have on both endothelial cells and pericytes, while pericyte-derived inflammatory molecules may also potentially have wider consequences for virally induced immune dysregulation. Disentangling these highly complex interactions will benefit from the development of multicomponent cultures and organoids. Ultimately, a better understanding of virus-pericyte interactions could inform new therapies and diagnostic approaches for viral diseases, as has already been or is being explored in the context of DENV [71, 72], HIV [83–88] and SARS-CoV-2 [80].
